# Comparison of the effects of pure vitamin E and combined conservative therapy for precancerous cervical lesions cultivated on a microfluidic chip

**DOI:** 10.1515/med-2026-1413

**Published:** 2026-06-17

**Authors:** Anđela Perić, Ana N. Mirić, Jelena V. Košarić, Nevena N. Milivojević, Marko N. Živanović, Miloš N. Milosavljević, Simona Protrka, Petar S. Arsenijević

**Affiliations:** Clinic of Gynecology and Obstetrics, University Clinical Center of Kragujevac, Kragujevac, Serbia; Institute for Information Technologies, University of Kragujevac, Kragujevac, Serbia; Department of Pharmacology and Toxicology, Faculty of Medical Sciences, University of Kragujevac, Kragujevac, Serbia; Department of Communication Skills, Ethics and Psychology, Faculty of Medical Sciences, University of Kragujevac, Kragujevac, Serbia; Department of Gynecology and Obstetrics, Faculty of Medical Sciences, University of Kragujevac, Kragujevac, Serbia

**Keywords:** vitamin E, microfluidic chip, precancerous cervical lesions, BAX, BCL2

## Abstract

**Objectives:**

We aimed to examine the effect of vitamin E on preinvasive cervical squamous epithelium lesions on a microfluidic chip model.

**Methods:**

The research was conducted on 60 women, previously determined for a biopsy of the cervix due to an abnormal screening cytological test. The control group consisted of patients who were not treated with vaginal tablets Ialuna^®^ or pure vitamin E. Samples were cultivated on a microfluidic chip, treated with Ialuna^®^ vaginal tablets containing vitamin E combined with other ingredients or pure vitamin E, and then relative gene expression of BAX and BCL2 genes was measured.

**Results:**

In tissues from women with chronic cervicitis, treatment with Ialuna^®^ vaginal tablets induced a significant increase in BAX expression and the BAX/BCL2 ratio, while no significant changes were observed in BCL2 expression. In LSIL tissues, Ialuna^®^ treatment resulted in a significant decrease in BCL2 expression, accompanied by increased BAX expression and an elevated BAX/BCL2 ratio. Conversely, pure vitamin E treatment did not produce significant changes in apoptotic markers in either group.

**Conclusions:**

This study has shown that conservative therapy is an important part of the treatment of cervical precancerous lesions and can potentially lead to diminishing radical interventions.

## Introduction

Cervical cancer remains one of the leading causes of morbidity and mortality among women worldwide, particularly in developing countries, where it accounts for the majority of deaths [1], 2]. Despite well-established screening programs and the proven role of high-risk human papillomavirus (HPV) infection in cervical carcinogenesis, the biological processes underlying malignant transformation are complex and not yet fully clarified. Besides viral infection, additional factors such as smoking, malnutrition, sexual behavior, and chronic inflammation contribute to oxidative stress, reduced apoptosis, and enhanced cellular proliferation, thereby promoting tumor development [3], [4], [5], [6].

Most cases of cervical cancer arise through preinvasive lesions, detectable by cytological screening (Pap test). While many of these lesions regress spontaneously, a significant proportion persist or progress, making their early detection and management essential [7], [8], [9], [10], [11]. Current experimental models for investigating cervical carcinogenesis, however, are largely based on conventional two-dimensional (2D) cultures or animal studies. These models provide valuable insights but have significant limitations, particularly in reproducing the complex three-dimensional microenvironment of cervical tissue and in predicting therapeutic responses [12].

Microfluidic “organ-on-chip” systems offer a promising alternative by integrating cellular bioengineering with controlled microenvironments that closely mimic *in vivo* physiology [13], [14], [15], [16]. This approach has already been successfully applied to study tissues such as lung, kidney, intestine, liver, and skin [17], [18], [19], [20], [21], [22], [23]. Yet, to date, dysplastic cervical tissue has not been explored within this platform, representing an important gap in the field [24]. Two-dimensional cultures fail to reproduce the complex cell-cell and cell-matrix interactions present in native cervical tissue. Ex vivo tissue biopsies, on the other hand, provide more physiologically relevant information but lack dynamic perfusion and are typically maintained only for short periods under static conditions. In contrast, the proposed microfluidic system allows for continuous perfusion of the culture medium and therapeutic agents, ensuring an adequate supply of nutrients that supports tissue viability over a longer period. Furthermore, the controlled microenvironment and spatial confinement within the chip more closely mimic *in vivo* physiological conditions compared to static culture systems. These features increase the reliability of the model for monitoring treatment response and cellular dynamics in dysplastic cervical tissue.

Antioxidants, including vitamin E (α-tocopherol), have been investigated as potential modulators of oxidative stress, apoptosis, and inflammation in various tissues. Their relevance in cervical carcinogenesis stems from the role of reactive oxygen species in DNA damage and cellular transformation [4], 12]. However, the effects of vitamin E on preinvasive cervical lesions remain insufficiently studied, particularly in advanced *in vitro* models that allow precise simulation of tissue microenvironments. Ialuna^®^ vaginal tablets contain hyaluronic acid (HA), *Centella asiatica* extract (gotu kola), *Matricaria chamomilla* plant extract (chamomile) and D,L α-tocopherol acetate (vitamin E).

HA is a glycosaminoglycan that binds large amounts of water, forming a hydrated gel layer on mucosal surfaces. This maintains tissue flexibility, supports barrier integrity, and reduces environmental stressors that can exacerbate oxidative damage. By improving epithelial barrier function and healing, HA may reduce ongoing irritative stimuli and support recovery of normal tissue architecture – potentially lowering the local pro-oxidative environment [25].

Centella Asiatica extract (Gotu Kola) has antioxidative, cytoprotective as well as anti-inflammatory effects. Centella’s triterpenoids and polyphenols search for ROS and upregulate endogenous antioxidant enzymes (like catalase and superoxide dismutase), lowering oxidative stress at the cellular level. It also reduces pro-inflammatory mediators (TNF-α, IL-6) that are often induced by oxidative stress, helping modulate chronic inflammation. In cell models, Centella extracts have been shown to reduce oxidative stress-induced apoptosis and cellular senescence caused by ROS. Therefore, by enhancing antioxidant defenses and lowering ROS production, Centella supports mucosal healing and resilience against oxidative injury [26].

*Matricaria chamomilla* (chamomile) plant extract contains flavonoids and terpenoids that scavenge free radicals and lower oxidative stress. It can inhibit pro-inflammatory pathways, reducing the inflammatory burden that often accompanies oxidative imbalance. Chamomile’s soothing and ROS-reducing properties can lower local inflammation and irritation of the vaginal mucosa, thereby decreasing oxidative damage from inflammatory cell activity [27].

Vitamin E (D, l-α-tocopherol acetate) is a lipid-soluble antioxidant that incorporates into cell membranes and protects polyunsaturated lipids from peroxidation by neutralizing lipid-based radicals. By preserving membrane integrity under oxidative attack, vitamin E helps maintain cellular function and prevents propagation of ROS-induced damage. Studies found that higher serum levels of vitamin E correlate with a lower prevalence of cervical dysplasia and CIN, and lower vitamin E levels have been observed in women with cervical intraepithelial neoplasia compared to controls [28].

High spontaneous regression rates and aggressive treatment followed by frequent complications led us to believe that conservative therapy was a better choice. We considered that the use of vitamin E combined with other elements locally could help lower oxidative stress in cervical tissue, regulate activity of proapoptotic and antiapoptotic genes BAX and BCL2, and encourage regression of these lesions even more, which would in the future reduce the need for aggressive treatment. Furthermore, a special interest was in the use of a new technology and a tissue that had not been studied on a microfluidic chip thus far.

Therefore, the aim of this study was to examine the effects of vitamin E and Ialuna^®^ vaginal tablets on preinvasive cervical squamous epithelial lesions cultured on a microfluidic chip model.

## Materials and methods

### Patients

The study included 60 women scheduled for cervical biopsy due to abnormal cytological screening results (Pap test).

The control group consisted of untreated samples, analyzed 24 h after cultivation and therefore referred to as the “24 h control group.” In addition, two experimental groups were established: the first was treated either with pure vitamin E or Ialuna^®^ vaginal tablets and analyzed 72 h after cultivation, while the second was treated under the same conditions and analyzed after 144 h. The analyzed material comprised 34 cases of chronic cervicitis that had initially been misdiagnosed as precancerous lesions during routine Pap smear screening, 20 cases of low-grade squamous intraepithelial lesions (LSIL), and 6 cases of high-grade squamous intraepithelial lesions (HSIL). Cervical biopsies were obtained with voluntary participation and signed informed consent between August 2023 and March 2024 at the Clinic of Gynecology and Obstetrics, University Clinical Center Kragujevac.

Histopathological verification was performed ex tempore to confirm that the specimens represented tissue with limited malignant potential. Subsequently, part of the biopsy material was processed to establish a primitive 3D culture on a microfluidic chip device. In 6 out of 20 LSIL samples and 14 out of 34 chronic cervicitis samples, cultivation on the chip was unsuccessful. This failure is most likely attributable to the intrinsic biological properties of cervical biopsy tissue, which are notoriously difficult to maintain *in vitro*. The complex tissue architecture limited cellular viability post-biopsy, while susceptibility to necrosis induced by ischemic stress and enzymatic degradation further hindered stable 3D culture formation. In addition, the mechanical and biochemical characteristics of the extracellular matrix may have negatively affected cellular adhesion and proliferation within the microfluidic system.

### Fabrication of microfluidic chip device

**Figure 1:**
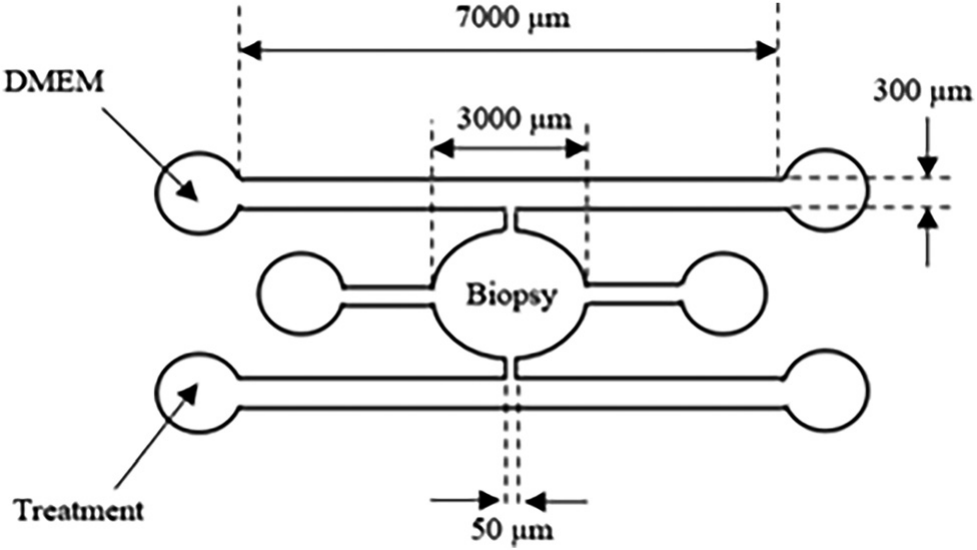
Schematic diagram of a microfluidic chip designed for the culture and treatment of a biopsy sample. In the central part, there is a chamber intended for placing a tissue sample. The chip has two main channels – upper and lower, where the upper one is connected to the medium inlet, while the lower one is connected to the treatment inlet. These channels enable simultaneous delivery of the nutrient medium and the therapeutic agent to the biopsy sample. The dimensions of the system are optimized for stable flow and efficient diffusion. The length of the main channels is 7,000 µm, the width is 300 µm, while the distance between the connection channels and the central chamber is 3,000 µm. The distance between the upper and lower channels in the biopsy zone is 50 µm, which enables a close interaction of the nutrient medium and the treatment with the tissue. At the ends of the channels there are reservoirs for fluid input and output, which ensures a continuous and controlled flow.

This is the house-made microfluidic chip device. The microfluidic chamber and channels were designed using CAD software. The device consists of three compartments, including a circular central chamber containing a hydrogel-based extracellular matrix (Matrigel^®^) with the tissue sample, as well as separate inlet and outlet ports. Surrounding the central chamber are perfusion channels designed to mimic the morphology of tumor microvasculature, which remain non-interconnected (Figure 1). The central chamber is intended for placement of the tissue sample. The chip features two main channels, an upper and a lower one. The upper channel is connected to the medium inlet, while the lower channel is connected to the treatment inlet, enabling the simultaneous delivery of nutrient medium and therapeutic agents to the biopsy sample. The system dimensions are optimized to ensure stable flow and efficient diffusion. The main channels are 7,000 µm in length and 300 µm in width, while the distance between the connecting channels and the central chamber is 3,000 µm. In the biopsy region, the distance between the upper and lower channels is 50 µm, allowing close interaction between the nutrient medium, the treatment, and the tissue. At the channel termini, reservoirs for fluid input and output are present, ensuring continuous and controlled flow.

The three-compartment geometry was selected to better recapitulate the structural and functional organization of the tumor microenvironment. Specifically, the central compartment allows the embedding of dysplastic cervical tissue or tumor cells within an extracellular matrix mimic, while the two adjacent lateral channels provide independent perfusion of culture medium and therapeutic agents. This design enables controlled diffusion of nutrients, oxygen, and drugs into the tissue compartment, as well as the possibility to generate chemical gradients across the construct. Comparable designs have been successfully implemented in tumor-on-chip models, where a central hydrogel or tissue chamber is flanked by perfusable channels to mimic vascular supply and drug delivery [29], 30].

Standard UV-photolithography and replica molding techniques were employed for mold fabrication. The microfluidic chip was constructed from poly (dimethylsiloxane) (PDMS – SYLGARD™ 184 Silicone Elastomer Kit), chosen for its optical transparency, which enables real-time imaging, immunocytochemistry, and subsequent harvesting of cells for gene and protein expression analysis. The photomask was produced by mask stereolithography (mSLA) 3D printing and subsequently post-processed by exposure to UV light for 4 h to ensure complete curing and dimensional stability. The chip was fabricated using PDMS. The material was prepared by mixing the pre-polymer and curing agent in a 10:1 ratio. The mixture was then poured into a mold and placed in a vacuum chamber to remove entrapped air bubbles. Finally, the mold was cured in an oven at 70 °C for at least 4 h to ensure complete polymerization. These channels were used to deliver culture medium and treatment agents. After pouring PDMS (10:1 w/w pre-polymer: cross-linker; Sylgard 184, Dow Corning) into the mold, the device was treated with O_2_ plasma to activate bonding and finalize the microfluidic chip [29].

**Figure 2:**
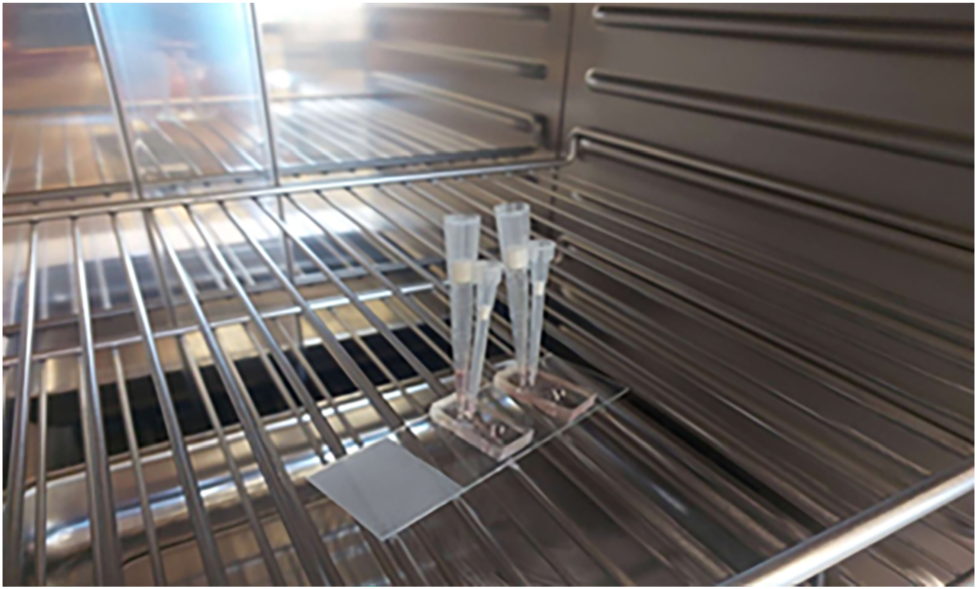
Microfluidic chip with a sample placed in a cell culture incubator. The chip is located on a glass substrate and is connected to reservoirs formed by conical tubes, which are used to supply the nutrient medium and the treatment. Placing the device in an in-cubator ensures controlled conditions of temperature, humidity and CO_2_ concentration, which is necessary to maintain the vitality of biopsy samples during the experiment. In this way, continuous supply of tissues with nutrients and simultaneous application of therapeutic agents in a physiologically relevant environment is enabled.

The chip is located on a glass substrate and is connected to reservoirs formed by conical tubes, which are used to supply the nutrient medium and the treatment. Placing the device in an incubator ensures controlled conditions of temperature, humidity and CO_2_ concentration, which is necessary to maintain the vitality of biopsy samples during the experiment. In this way, continuous supply of tissues with nutrients and simultaneous application of therapeutic agents in a physiologically relevant environment is enabled (Figure 2).

### Treatment of samples

Samples were randomly assigned into two groups. The first group was treated with Ialuna^®^ vaginal tablets (IBSA Farmaceutici Italia), which contain vitamin E in the form of D,L-α-tocopherol acetate (1 g), as well as hyaluronic acid, Centella Asiatica extract, and Matricaria Chamomilla. The tablets were dissolved in a water bath at 37 °C, and the resulting solution was applied to the samples. The second group was treated with pure vitamin E extract. Samples in the 24-h cohort remained untreated and served as controls. Total RNA was isolated after 24, 72, and 144 h. One vaginal tablet was dissolved in 100 mL of PBS in a water bath at a temperature of 37 °C. Each sample was treated with 200 mL of solution.

### Isolation of total RNA

#### Tissue homogenization

Tissue samples were homogenized in a metal ball homogenizer at maximum speed three times for 30 s and centrifuged at 500 rpm for 5 min at room temperature to remove tissue debris.

#### Isolation

Total RNA was extracted using the standard TRIzol^®^ protocol. Following chloroform addition and centrifugation at 15,000 RCF for 15 min at 4 °C, three phases were obtained: an upper aqueous phase containing RNA, a lower red phase with proteins, and an interphase containing DNA in the form of a white ring. After washing with isopropanol and ethanol and drying on a thermoblock, the RNA was dissolved in 30 µL of sterile water for injection. RNA concentration was measured using a Multiskan SkyHigh UV/VIS spectrophotometer at 260 nm, while purity was assessed by the 260/230 nm and 260/280 nm absorbance ratios [27]. RIN values were not determined due to the unavailability of a Bioanalyzer. Instead, RNA integrity was ensured by immediate processing of freshly isolated RNA directly into cDNA synthesis and qPCR analysis. All qPCR amplicons were 150–180 bp, single products were confirmed by melt-curve analysis.

### Reverse transcription

Total RNA was reverse-transcribed into complementary DNA (cDNA) using the High Capacity cDNA Reverse Transcription Kit (Applied Biosystems by Thermo Fisher Scientific) on a Multiskan Optimax (Labnet International), in accordance with the manufacturer’s instructions. The reverse transcription reaction was performed in a total volume of 20 µL, containing 10 µL of 2 × RT master mix into each well and 10 µL of RNA sample. The reaction proceeded in multiple steps. Primer annealing was performed at 25 °C for 10 min, and reverse transcription was carried out at 37 °C for 120 min, followed by a final incubation at 85 °C for 5 min to terminate the reaction. The samples were then cooled to 4 °C. The resulting cDNA, which is more stable than RNA, was subsequently stored at −80 °C until further analysis.

### Relative gene expression

Quantitative polymerase chain reaction (qPCR) assays were performed to determine relative gene expression according to laboratory protocols mainly based on MIQE Guidelines [31], in accordance with Good Laboratory Practice (GLP) principles for PCR detection and sample-centric laboratory manipulation. We determined relative expression levels of apoptosis-related genes (BCL-2 and BAX), with β-actin serving as the housekeeping gene (Table 1). Reactions were carried out using SYBR™ Green PCR Master Mix (Applied Biosystems by Thermo Fisher Scientific) on a Mic Real-Time PCR Cycler, following the manufacturer’s instructions [32], 33]. Cycling conditions were initial denaturation at 95 °C for 10 min, followed by 40 cycles of 95 °C for 15 s, 60 °C for 60 s (annealing/extension), and 72 °C for 30 s. A melt-curve analysis (72–95 °C, 0.3 °C/s) was performed at the end of each run to confirm specificity. All reactions were performed in technical triplicates, and negative controls without template (NTC) were included (Table 2). Relative expression levels were calculated using the 2−ΔΔCt method described by Livak and Schmittgen [34].

**Table 1:** Primers for qPCR.

Gene	Primer sequence
*Bcl – 2*	F 5′- gat​aac​gga​ggc​tgg​gat​gc -3′
	R 5′- gac​ttc​act​tgt​ggc​cca​gat -3′
*Bax*	F 5′- gct​tca​ggg​ttt​cat​cca​gga -3′
	R 5′- caa​tca​tcc​tct​gca​gct​cca -3′
*β-actin*	F 5′- ctc​acc​ctg​aag​tac​ccc​atc -3′
	R 5′- agg​tct​caa​aca​tga​tct​ggg -3′

**Table 2:** qPCR target information.

Target name	Abbreviation	Accessione number/locus	Amplicon length	Secondary structures
B-cell CLL/lymphoma 2	Bcl-2	NM_000633.3	154 bp	No
Bcl-2-associated X protein	Bax	NM_138761.4	164 bp	No
Beta-actin (Housekeeping)	β-actin	NM_001101.5	1812 bp	Very weak

Tables 1 and 2 show genes of interest and corresponding primers.

### Statistical analysis

Statistical analyses were performed using SPSS software, version 18 (SPSS Inc., Chicago, IL, USA). Socio-demographic and clinical data were summarized using descriptive statistics (frequencies and relative values). The chi-square test was used to compare distributions of socio-demographic and clinical characteristics among the three groups (chronic cervicitis, LSIL, HSIL). Gene expression data were first tested for normality using the Shapiro–Wilk test. Since the data were not normally distributed, nonparametric Friedman and Wilcoxon tests with Bonferroni correction were applied to assess the effects of pure vitamin E and Ialuna^®^ tablets over time. To compare differences between Ialuna^®^ and pure vitamin E, the Mann–Whitney U test was used. A p-value of less than 0.05 was deemed statistically significant in the Mann-Whitney and Friedman tests. In contrast, for the post hoc Wilcoxon test with Bonferroni correction, we used a p-value of 0.0167 as the threshold, since this test involved comparisons among three groups: the control and two experimental groups.

### Ethics approval and consent to participate

The study was conducted in accordance with the Declaration of Helsinki, and approved by the Ethics Committee of University Clinical Center Kragujevac (protocol code No. 01/23–131 and date of approval 10.04.2023.).

Informed consent was obtained from all individuals included in this study, or their legal guardians or wards.

## Results

### The effects of vitamin E on the gene expression

#### Chronic cervicitis group

In tissues obtained from women with chronic cervicitis treated with either Ialuna^®^ vaginal tablets or pure vitamin E, no significant differences were observed in BCL2 expression over time (p=0.819 and p=0.247, respectively). A statistically significant increase in BAX expression was detected in the Ialuna^®^ group (p=0.002), but not in the pure vitamin E group (p=0.074). Similarly, a significant rise in the BAX/BCL2 ratio was observed in the Ialuna^®^ group (p=0.001), while no significant change was noted with pure vitamin E (p=0.074).

#### LSIL group

In LSIL tissues treated with Ialuna^®^ tablets, significant changes were observed in BCL2 expression (p=0.016), BAX expression (p=0.013), and the BAX/BCL2 ratio (p=0.002). Specifically, BCL2 expression decreased while BAX expression increased over time. In contrast, no significant changes were observed in the pure vitamin E group (BCL2: p=0.449; BAX: p=0.247; BAX/BCL2 ratio: p=0.819).

#### HSIL group

In HSIL tissues treated with Ialuna^®^ tablets, no significant changes were detected in BCL2 (p=0.449) or BAX expression (p=0.165). However, a significant change in the BAX/BCL2 ratio was observed over time (p=0.016). Only one HSIL sample was treated with pure vitamin E, precluding statistical analysis of this group.

The effects of vitamin E on apoptosis-related gene activity are summarized in Table 3.

**Table 3:** The effects of vitamin E on the activity of genes associated with apoptosis.

Chronic cervicitis group					
Intervention	24 h control group Mean ± SD (median; interquartile range)^c^	72 h Mean ± SD (median; interquartile range)	144 h Mean ± SD (median; interquartile range)	Friedman test	Post-hoc Wilcoxon test with bonferroni correction
**Expression of *BCL2* gene**					

Ialuna^®^ vaginal tablets	4.16 ± 4.29 (3.67; 3.88)	4.27 ± 8.04 (0.93; 2.76)	6.60 ± 11.13 (1.85; 5.36)	χ2=0.400 p=0.819	/
Pure vitamin E	9.12 ± 13.19 (5.18; 31.74)	1.49 ± 1.64 (0.92; 3.03)	3.63 ± 5.26 (1.90; 7.74)	χ2=2.800 p=0.247	/

**Expression of *BAX* gene**					

Ialuna^®^ vaginal tablets	3.76 ± 10.36 (0.37; 1.21)	2.81 ± 3.10 (1.93; 2.73)	41.37 ± 82.14 (9.85; 21.89)	χ^2^=13.000 **p=0.002** ^**a**^	24 h vs. 72 h: Z=−2.919, **p=0.004** ^**a**^
					24 h vs. 144 h: Z=−3.124, **p=0.002** ^**a**^
					72 h vs. 144 h: Z=−2.542, **p=0.011** ^**a**^
Pure vitamin E	0.83 ± 0.81 (0.64; 1.19)	11.63 ± 19.67 (3.78; 24.61)	7.85 ± 6.70 (7.63; 11.45)	χ^2^=5.200 p=0.074	/

***BAX*/*BCL2* ratio**					

Ialuna^®^ vaginal tablets	0.54 ± 0.74 (0.13; 1.15)	3.37 ± 4.27 (1.22; 5.17)	42.90 ± 103.48 (4.18; 7.93)	χ2=14.286 **p=0.001** ^**a**^	24 h vs. 72 h: Z=−2.229, p=0.026
					24 h vs. 144 h: Z=−3.296, **p=0.001** ^**a**^
					72 h vs. 144 h: Z=−1.538, p=0.124
Pure vitamin E	0.37 ± 0.40 (0.32; 0.68)	3.94 ± 3.14 (4.11; 5.35)	93.26 ± 200.83 (4.58; 451.87)	χ^2^=5.200 p=0.074	/

**LSIL group**					

**Expression of *BCL2* gene**					

Ialuna^®^ vaginal tablets	14.11 ± 27.16 (4.81; 10.20)	2.52 ± 4.39 (0.92; 2.56)	2.15 ± 2.73 (1.18; 2.76)	χ2=8.222 **p=0.016** ^**a**^	24 h vs. 72 h: Z=−1.836, p=0.066
					24 h vs. 144 h: Z=−1.955, p=0.051
					72 h vs. 144 h: Z=−0.059, p=0.953
Pure vitamin E	0.27 ± 0.23 (0.22; 0.45)	4.85 ± 9.76 (0.56; 11.15)	4.05 ± 5.07 (2.82; 7.16)	χ2=1.600 p=0.449	/

**Expression of *BAX* gene**					

Ialuna^®^ vaginal tablets	3.84 ± 8.28 (0.90; 2.63)	3.58 ± 3.96 (1.65; 4.43)	19.86 ± 39.85 (3.62; 16.43)	χ2=8.667 **p=0.013** ^**a**^	24 h vs. 72 h: Z=−0.770, p=0.441
					24 h vs. 144 h: Z=−1.481, p=0.139
					72 h vs. 144 h: Z=−2.666, **p=0.008** ^**a**^
Pure vitamin E	5.36 ± 6.17 (2.18; 11.73)	7.09 ± 7.36 (5.62; 14.10)	21.64 ± 23.05 (18.66; 42.33)	χ2=2.800 p=0.247	/

***BAX*/*BCL2* ratio**					

Ialuna^®^ vaginal tablets	1.01 ± 1.84 (0.19; 1.53)	5.67 ± 6.30 (1.90; 10.48)	9.63 ± 9.40 (7.11; 15.52)	χ2=12.667 **p=0.002** ^**a**^	24 h vs. 72 h: Z=−2.073, p=0.038
					24 h vs. 144 h: Z=−2.666, **p=0.008** ^**a**^
					72 h vs. 144 h: Z=−2.073, p=0.038
Pure vitamin E	32.97 ± 54.55 (8.87; 71.57)	15.03 ± 13.85 (17.40; 27.27)	6.76 ± 7.27 (5.30; 11.40)	χ2=0.400 p=0.819	/

**HSIL group**					

**Intervention**	**24 h** **Mean ± SD (median; interquartile range)**	**72h** **Mean ± SD (median; interquartile range)**	**144 h** **Mean ± SD (median; interquartile range)**	Friedman test	Post-hoc Wilcoxon test

**Expression of *BCL2* gene**					

Ialuna^®^ vaginal tablets	4.28 ± 4.66 (2.30; 7.53)	4.38 ± 7.13 (1.40; 10.19)	3.51 ± 4.00 (1.93; 7.75)	χ2=1.600 p=0.449	/
Pure vitamin E				/^b^	/

**Expression of *BAX* gene**					

Ialuna^®^ vaginal tablets	10.49 ± 22.80 (0.24; 25.76)	17.74 ± 29.90 (0.76; 43.23)	21.66 ± 21.75 (7.86; 40.68)	χ2=3.600 p=0.165	/
Pure vitamin E					

***BAX*/*BCL2* ratio**					

Ialuna^®^ vaginal tablets	3.99 ± 8.97 (0.34; 6.20)	15.41 ± 20.73 (8.13; 27.49)	79.46 ± 111.50 (13.85; 198.91)	χ2=8.333 **p=0.016** ^**a**^	24 h vs. 72 h: Z=−1.572, p=0.116
					24 h vs. 144 h: Z=−2.201, p=0.028
					72 h vs. 144 h: Z=−1.363, p=0.173
Pure vitamin E					

SD, standard deviation; ^a^statistically significant; ^b^insufficient samples for statistical analysis.

**Figure 3:**
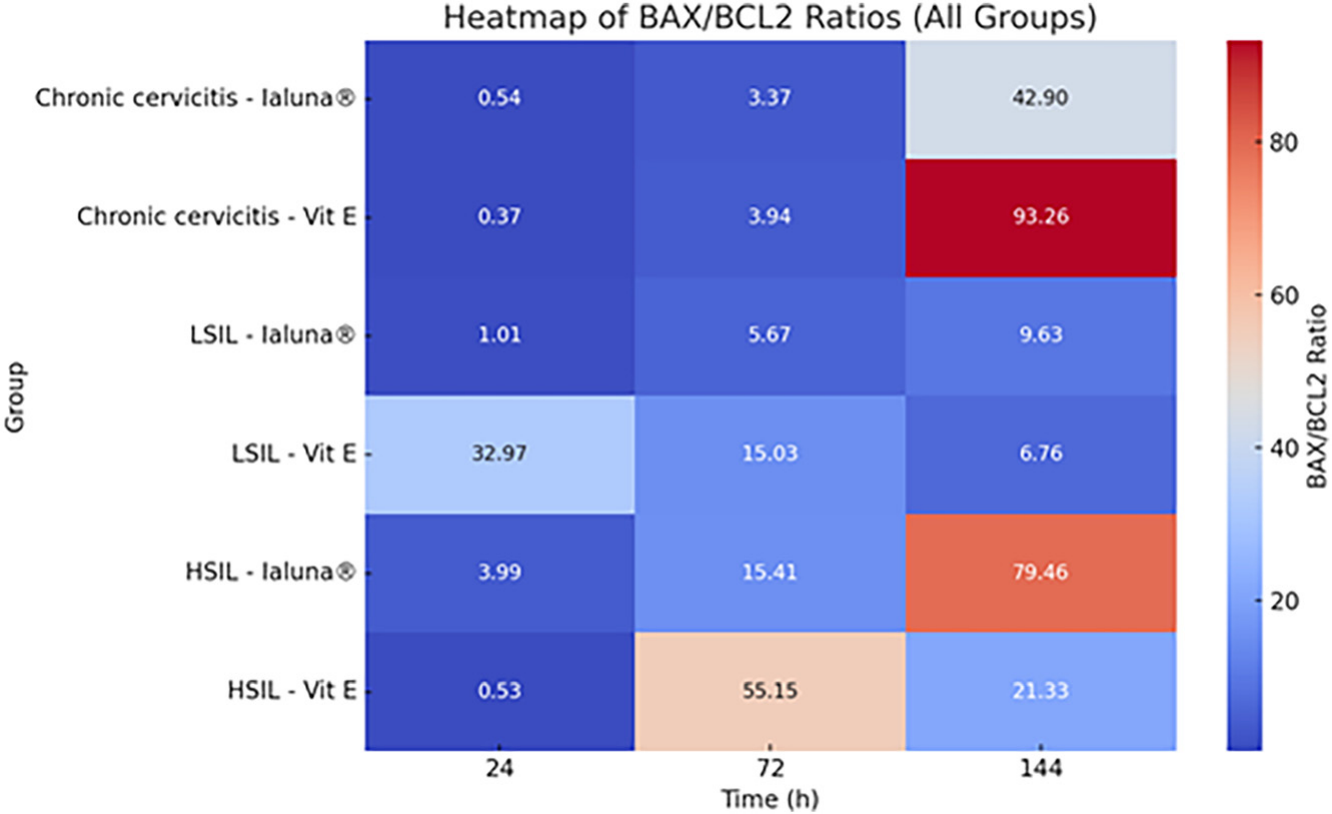
Heatmap representation of Bax/Bcl-2 ratios across cervical tissue groups (chronic cervicitis, LSIL, HSIL) treated with Ialuna^®^ vaginal tablets or pure vitamin E at 24 h (control group), 72 h, and 144 h. numeric values represent mean ratios for each condition. Warmer colors indicate higher Bax/Bcl-2 ratios, corresponding to stronger pro-apoptotic signaling.

To facilitate visual interpretation of the temporal and group-specific changes in apoptotic activity, we generated a heatmap summarizing the Bax/Bcl-2 ratios across all groups and time points (Figure 3). This graphical representation complements the tabulated values by highlighting relative differences and trends among the treatment groups.

### Differences in the effects of the Ialuna^®^ vaginal tablets and pure vitamin E on gene expression

The Mann-Whitney U test revealed no statistically significant differences between the effects of Ialuna^®^ vaginal tablets and pure vitamin E on the expression of BCL2 gene, BAX genes, or the BAX/BCL2 ratio in any of the clinically tested groups (chronic cervicitis, LSIL, and HSIL), either after 72 h or 144 h of exposure (Table 4).

**Table 4:** Differences in the effects of the Ialuna^®^ vaginal tablets and pure vitamin E on gene expression.

Parameter	Group	Ialuna^®^ vaginal tablets Mean ± SD (median; interquartile range)	Pure vitamin E Mean ± SD (median; interquartile range)	Mann-Whitney U test
**72 h**				

Expression of *BCL2* gene	Chronic cervicitis	4.27 ± 8.04 (0.93; 2.76)	1.49 ± 1.65 (0.92; 3.03)	U=29.000 p=0.497
	LSIL	2.52 ± 4.39 (0.92; 2.56)	4.85 ± 9.76 (0.56; 11.15)	U=20.000 p=0.797
	HSIL	4.38 ± 7.13 (1.40; 10.19)	0.127^a^	U=2.000 p=1.000
Expression of *BAX* gene	Chronic cervicitis	2.81 ± 3.10 (1.93; 2.73)	11.63 ± 19.67 (3.78; 24.61)	U=22.000 p=0.257
	LSIL	3.59 ± 3.96 (1.65; 4.43)	7.09 ± 7.36 (5.62; 14.10)	U=21.000 p=0.898
	HSIL	17.74 ± 29.90 (0.76; 42.23)	6.990^a^	U=2.000 p=1.000
***BAX*/*BCL*** *2* ratio	Chronic cervicitis	3.37 ± 4.27 (1.23; 5.17)	3.82 ± 3.60 (3.14; 6.79)	U=23.000 p=0.645
	LSIL	5.67 ± 6.30 (1.90; 10.48)	15.03 ± 13.84 (17.40; 27.27)	U=15.000 p=0.364
	HSIL	7.46 ± 7.95 (5.11; 14.54)	55.15^a^	U=0.000 p=0.333

**144 h**				

Expression of *BCL2* gene	Chronic cervicitis	6.60 ± 11.13 (1.84; 5.36)	3.62 ± 5.26 (1.90; 7.75)	U=36.000 p=0.933
	LSIL	2.15 ± 2.73 (1.18; 2.76)	4.05 ± 5.07 (2.82; 7.16)	U=15.000 p=0.364
	HSIL	3.51 ± 4.00 (1.93; 7.75)	0.213^a^	U=2.000 p=1.000
Expression of *BAX* gene	Chronic cervicitis	41.37 ± 82.14 (9.85; 21.89)	9.65 ± 7.44 (8.18; 16.22)	U=42.000 p=0.850
	LSIL	19.85 ± 39.85 (3.62; 16.43)	21.64 ± 23.05 (18.66; 42.33)	U=20.000 p=0.797
	HSIL	21.65 ± 21.75 (7.86; 40.68)	4.547^a^	U=1.000 p=0.667
***BAX*/*BCL*** *2* ratio	Chronic cervicitis	42.90 ± 103.48 (4.18; 7.93)	78.82 ± 183.07 (5.60; 117.27)	U=33.000 p=0.494
	LSIL	9.63 ± 9.40 (7.11; 15.52)	6.76 ± 7.27 (5.30; 11.40)	U=17.000 p=0.518
	HSIL	91.08 ± 120.53 (6.36; 216.47)	21.33^a^	U=2.000 p=1.000

SD, standard deviation; ^a^insufficient samples for statistical analysis.

## Discussion

In the present study we showed the use of vaginal tablets resulted in a significant increase in the BAX/BCL2 ratio in tissues obtained from women with chronic cervicitis and LSIL lesions, whereas treatment with pure vitamin E did not produce a statistically significant effect in these groups. In the HSIL group, a statistically significant alteration in the BAX/BCL2 ratio was observed following vaginal tablet administration. However, due to the limited number of samples treated with pure vitamin E, a reliable comparative analysis for this group could not be performed.

Oxidative stress plays a central role in cervical carcinogenesis. The accumulation of reactive oxygen species (ROS) damages cervical epithelial cells, activating multiple signaling pathways that may lead to mutations or trigger cell death via apoptosis or necrosis [35], [36], [37].

Vitamin E is a potent antioxidant that captures free radicals and prevents oxidative chain reactions [4], 12]. Reduced vitamin E levels have been documented in cervical cancer patients. Based on this evidence, we compared the effects of Ialuna^®^ vaginal tablets (containing vitamin E in combination with hyaluronic acid, Centella Asiatica extract, and Matricaria Chamomilla) and pure vitamin E on apoptosis regulation in cervical tissues of varying histopathological status (chronic cervicitis, LSIL, HSIL).

The combined formulation of Ialuna^®^ tablets possesses strong synergistic potential, as each component exhibits antioxidant properties. The BAX/BCL2 ratio was selected as the key parameter, given its role as a reliable indicator of apoptotic activity. Members of the BCL2 family are pivotal regulators of tumor progression and intrinsic apoptosis triggered by mitochondrial dysfunction [38], 39]. Thus, the balance between pro-apoptotic (BAX) and anti-apoptotic (BCL2) proteins is critical in determining cell fate [40].

Numerous *in vitro* studies indicate that different forms of vitamin E can significantly influence the expression of genes and proteins from the Bcl-2 family, thereby modulating apoptosis in tumor cells. For example, γ-tocopherol and especially γ-tocotrienol showed a strong pro-apoptotic effect through an increase in Bax expression and a relative decrease or weaker increase in Bcl-2, resulting in an increase in the Bax/Bcl-2 ratio and the induction of apoptosis in leukemic cell lines [41]. Similarly, δ-tocotrienol activated the intrinsic apoptotic pathway in melanoma cells and led to a marked shift in the balance towards a pro-apoptotic response, whereby an increase in Bax was detected in one line, while in the other the dominant effect was a decrease in Bcl-2, which in both cases resulted in a favorable increase in the Bax/Bcl-2 ratio [42]. In addition to pure isomers of vitamin E, combined formulations, such as resveratrol in synergy with vitamin E, showed a similar effect – in colorectal cells, this combination caused a significant increase in Bax with a simultaneous decrease in Bcl-2, which further enhanced the pro-apoptotic signal [43]. More recent results, such as the work of Diyarkojouri et al. [44], show that vitamin E can simultaneously increase both Bax and Bcl-2 [44]. The final effect depends on which gene is more strongly activated, so the response can be different depending on the type of cells and the applied dose. Overall, the data indicate that vitamin E, in various forms and combinations, most often leads to a shift in the balance in favor of apoptosis through modulation of Bax and Bcl-2, although specific expression patterns are context-dependent.

Our findings demonstrated that Ialuna^®^ tablets significantly increased the Bax/Bcl-2 ratio in chronic cervicitis (p=0.001) and LSIL tissues (p=0.002), whereas pure vitamin E had no significant effect. Vitamin E’s antioxidant effects can be complementary with plant-derived polyphenols, creating a broader defense against ROS. Together, these substances create a multi-level protective effect. Hyaluronic acid hydrates and protects the vaginal epithelium, reducing mechanical stress and dehydration-induced ROS. Centella and Chamomile extracts provide direct antioxidant scavenging of free radicals while also down-regulating inflammation triggered by oxidative stress, interrupting the cycle of ROS production and tissue damage whereas vitamin E stabilizes membranes and prevents lipid peroxidation [25], [26], [27], [28].

This combination targets both the cause (ROS) and the consequences (inflammation, cellular damage) of oxidative stress. In HSIL, Ialuna^®^ tablets induced a significant increase in the Bax/Bcl-2 ratio (p=0.016), but the limited sample size in the pure vitamin E group prevented meaningful comparison. Importantly, in the LSIL group, a significant increase in the Bax/Bcl-2 ratio was already evident after 24 h (p=0.007), suggesting that the additional active components in Ialuna^®^ exert synergistic effects with vitamin E, thereby enhancing pro-apoptotic signaling.

These results highlight the therapeutic potential of formulations targeting oxidative stress in cervical precancerous lesions, supporting apoptosis as a favorable outcome over necrosis. Moreover, the time-dependent effects observed – particularly the progressive changes at 72 and 144 h – suggest sustained biological activity of the Ialuna^®^ tablets, emphasizing the importance of continuous application.

The main limitation of this study is the relatively small sample size, especially in the HSIL group. Future research with larger cohorts is required to confirm these findings, explore the specific molecular mechanisms of individual tablet components, and establish their clinical utility.

## Conclusions

This study provides preliminary evidence that vaginal tablets containing vitamin E, due to the synergistic action of their additional components, represent a promising conservative therapeutic approach for cervical lesions, particularly those of low malignant potential (LSIL). By enhancing apoptotic activity, these tablets may, in the future, contribute to the enhancement of conservative treatment of early stages of precancerous lesions and thus preserve the fertility potential of the treated patients [45].
